# Disordered eating among Arab and Jewish youth in Israel: the role of eating dinner with the family

**DOI:** 10.1186/s13584-020-00388-z

**Published:** 2020-06-10

**Authors:** Roni Elran-Barak, Michal Bromberg, Tal Shimony, Rita Dichtiar, Nisim Mery, Lesley Nitsan, Lital Keinan-Boker

**Affiliations:** 1 University of Haifa, School of Public Health, 199 Aba Khoushy Ave. Mount Carmel, Haifa, Israel; 2 Israel Center for Disease Control, Israel Ministry of Health, Ramat Gan, Israel; 3 Tel Aviv University, School of Public Health, Tel Aviv, Israel

**Keywords:** Disordered eating, Arab, Jewish, Youth, Ethnic minority, Family meals, Israel

## Abstract

**Background:**

Disordered eating (DE), defined as unhealthy eating attitudes and behaviors, is considered a major public health problem among adolescents. Nevertheless, rates of DE among Arab and Jewish adolescents in Israel are still unknown. Furthermore, while previous studies have highlighted the role of frequent family meals as a protective factor against DE, studies examining home family dinners relative to other common dinner options (e.g., eating at home alone, eating out of the home, not eating dinner at all) are largely unavailable. We sought to use representative data of middle and high-school children in Israel in order to identify rates of DE among Arabs and Jews, while examining the relations of home family dinners (vs. other dinner options) with DE.

**Methods:**

A nationally representative school-based survey of 4926 middle and high-school children (11–19 years old) was conducted during 2015–2016. Participants indicated where and with whom they had eaten dinner the day before. The 5-item SCOFF questionnaire was used (> 2 affirmative items were considered a likely case of DE). Height and weight were measured by personnel.

**Results:**

DE was more prevalent among girls (29.7%) relative to boys (12.2%), Arabs (25.1%) relative to Jews (19.5%), and older (25.3%) relative to younger (17.6%) adolescents. Arabs were more likely to eat dinner at home with parents/family (chi^2^ = 10.75, *p* = .001), or not to eat dinner at all (chi^2^ = 63.27, *p* < .001), while Jews were more likely to eat dinner alone (chi^2^ = 5.37, *p* = .021) or to eat dinner out of the home (chi^2^ = 67.65, *p* < .001). Logistic regressions (stratified by ethnicity and adjusted for gender, age, weight) revealed that family dinners acted as a protective factor against DE, relative to eating out of the home or relative to not eating dinner at all among both ethnic groups, and relative to eating dinner alone among Arabs.

**Conclusion:**

There are differences between Arab and Jewish adolescents in terms of rates of yesterday’s family dinners and DE. Given that eating dinner with the family was linked with lower rates of DE, possible interventions to reduce DE may include educating parents of both Arab and Jewish adolescents regarding the importance of family meals.

## Introduction

Over the past decade, there has been an increasing interest in disordered eating (DE), defined as unhealthy eating attitudes and behaviors that include, for example, preoccupation with body weight and shape, food restriction, fasting, binge eating, and vomiting [1–3]. Disordered eating is a major public health problem among adolescents [4] because of its high prevalence and its potentially serious physical and psychological consequences, which include malnutrition, weight gain, depressive symptoms, and low self-esteem [5]. In the USA, as many as 25% of adolescents report weight dissatisfaction, and almost 40% of adolescent males and 50% of adolescent females engage in unhealthy weight-control behaviors [6]. Disordered eating has been documented across gender, ethnicity, race, and sexual orientation [7]. Nevertheless, studies about DE among ethnic minorities in Western countries are relatively scarce.

Studying the prevalence and risk factors of DE among ethnic minorities in Western countries is important because DE is a bio-psychosocial condition [8] that is influenced by sociocultural factors and norms (e.g., the thin-ideal standard of beauty) [9]. Studies about DE in the USA have suggested a high prevalence of DE among youth of ethnic minorities [1]. Both Hispanic and American Indian female youth in the USA have reported a higher prevalence of DE in comparison to that reported by white female youth. Likewise, Hispanic, American Indian, black, and Asian male youth in the USA have reported a higher prevalence of DE in comparison to that reported by white male youth [10, 11]. Despite these findings about the alarming rates of DE among minority youth in the USA, there is only a limited amount of empirical data describing DE among minority youth around the world. The studies that have been conducted about DE outside of the USA (e.g., in Finland [12], Greece [13], and Norway [14]) used small sample sizes that did not allow for an accurate assessment of prevalence or risk factors among adolescents of ethnic minorities. Therefore, in the current study, we sought to examine the prevalence of DE in a large representative sample of Jewish and Arab middle and high-school children in Israel.

Israel is a multicultural society with various ethnic and religious groups [15]. About one fifth of Israel’s population comprises Arabs; of them, about 85% are Muslims and the rest are mainly Christians and Druze [16]. A national survey conducted in 2014 suggested that up to 25% of all youth in Israel reported dieting for weight loss [17], in comparison to only about 15% of youth in Europe. Another Israeli study from 2008 focused on female youth alone and suggested that nearly one third of girls in Israel reported at least two different forms of DE behaviors [18]. Latzer et al. [19–21], who have studied Arab youth in Israel, revealed a high prevalence of DE among this minority group, and suggested that Christian Arabs had significantly lower DE scores in comparison to Moslem Arabs and Druze. Nevertheless, representative studies comparing rates of DE across Jewish and Arab girls and boys in Israel are still largely unavailable [15, 21].

Regular family meals contribute to youths’ emotional and behavioral development [22]. Various studies have suggested that frequent family meals act as a protective factor against DE [23, 24], and also play an important role in promoting positive dietary intake and healthy eating patterns [25]. For example, prior studies found that youth who reported more frequent family meals had higher intakes of fruit, vegetables, and key nutrients, and lower intakes of soft drinks and saturated fat [26]. In addition, studies conducted among American [27] and British [28] adolescents suggested that girls who reported infrequent family meals had more extreme weight control behaviors (e.g., self-induced vomiting, use of laxatives) relative to girls who reported frequent family meals. Nevertheless, binge-eating was found to be linked with emotionally negative family-meal experiences and not with family meal frequency per se. Furthermore, most of the literature about family meals has focused on the impact of family meal frequency on DE [24], while studies looking at the impact of family dinners versus other dinner options (e.g., eating at home alone, eating out of the home, not eating at all) on DE are scarce.

Finally, despite the growing literature about family meals and DE, there is a lack of studies concentrating on family meals among ethnic minorities around the world and on the associations between family meals and DE across different ethnic groups. Studies conducted in the USA have suggested that family meals are more common among Asian Americans in comparison to other ethnic/racial groups [27], but differences in patterns of family meals among other ethnic minorities around the world (e.g., Arab youth in Israel) are largely unidentified.

Based on the literature review, the specific aims of this study were:

1. To describe the current prevalence of DE among Jewish and Arab adolescents in Israel.
2. To describe dinner patterns (i.e., eating at home with parents/family vs. eating at home alone, eating out of the home, or not eating dinner at all) among Jewish and Arab adolescents in Israel.
3. To examine whether eating dinner at home with parents/family members (vs. at home alone, out of the home, or not eating dinner at all) can act as a protective factor against DE among Jewish and Arab adolescents in Israel.

We hypothesized that both family dinners and DE would be more prevalent among Arabs relative to Jews, and that family dinners (vs. other dinner routines) would be linked with less DE. Findings from the current study will help professionals in the field to identify risk and protective factors for DE so that specific interventions and prevention programs can be tailored for both parents and youth of different ethnicities.

## Methods

The 2nd MABAT youth survey is one of a series of national health and nutrition surveys carried out by the Israeli Ministry of Health [29]. It is a cross-sectional, nationally representative, school-based study of adolescents in grades 7 to 12 (ages 11–19 years), and it was carried out from May 2015 to March 2016. A former survey aimed at the same age group was carried out in 2003–4 [18].

### Sampling and study population

The sample was drawn randomly from the Ministry of Education’s school list, with permission, using a cluster sampling method and ensuring representation of Israeli youth by population group (Jewish/Arab), framework (state/state religious), school level (middle school: grades 7–9; high school: grades 10–12), and welfare level (low/medium/high). The welfare level is a measure of the socioeconomic status (SES) in the area in which the school is located. Schools from the ultra-Orthodox (Haredi) sector were not included, nor were independent, private, or boarding schools [29].

The interviewers arrived at the classrooms, explained to the students about all parts of the survey, and distributed the self-administered questionnaire in Hebrew or Arabic.

### Anthropometric measurements

All adolescents who consented were measured for height and weight in a standardized and objective manner by trained personnel. Each measurement was performed twice and an average of the two measurements was used. If a third measurement was taken (due to a difference of more than 1 kg in weight or more than 0.5 cm in height), the average of the two measurements with the closest values was used. **Body mass index** (BMI) was calculated by kg/m^2,^ and BMI percentiles were calculated based on the age- and sex-specific WHO percentiles (http://www.who.int/growthref/who2007_bmi_for_age/en/). The categories that we used were: underweight (BMI percentile< 3%), healthy weight (3% ≤ BMI percentile< 85%), overweight (85% ≤ BMI percentile< 97%), and obese (BMI percentile≥97%) [30].

### The questionnaire

The self-administered questionnaire included a variety of questions on lifestyle and health habits [29]. The original questionnaire was developed in Hebrew and then translated into Arabic, for use within the Arab, Bedouin, and Druze sectors. Back-translation was carried out to ensure correct understanding and meaning. Classification as Jewish or Arab sector was determined by the school’s affiliated sector according to the Ministry of Education. Among Arab adolescents, 88% self-reported that they were Muslim, 5.3% self-reported that they were Christian, and 6.6% self-reported that they were Druze. SES was defined according to the welfare level of the school, an ecological index determined by the Ministry of Education based on the location of the school.

Presence of DE was determined by the SCOFF [31], a validated screening tool for DE among youth [32]. The SCOFF was originally developed as a screening tool for eating disorders. However, a recent meta-analysis [32] which looked at the diagnostic test characteristics of the SCOFF suggested that it was difficult to draw conclusions about the appropriateness of using the SCOFF to screen for eating disorders. Therefore, we decided to rely on recent studies among adolescents [33, 34] and adults [35] which used the SCOFF as a screening tool for DE, and not for eating disorder diagnoses such as anorexia nervosa or bulimia nervosa. The SCOFF includes five yes/no questions: (1) Do you make yourself **Sick** because you feel uncomfortably full? (2) Do you worry you have lost **Control** over how much you eat? (3) Have you ever lost more than **One** stone (=6.35 kg) in weight over a three-month period? (4) Do you believe yourself to be **Fat** when others say you are too thin? (5) Would you say that **Food** dominates your life? As suggested by Morgan et al. [31], each “yes” answer received one point, and a total score of more than two affirmative responses was considered to be a likely case of DE. In addition, the five different DE behaviors that appear in the SCOFF items were presented separately [36]. These five questions were worded in our questionnaire as they appear in the original SCOFF questionnaire, with the exception of question number three; specifically, the weight loss figure was reduced to 3 kg, from 6.35 kg, to allow for the generally lower body weights of adolescents as compared to that of adults. This exact same SCOFF questionnaire was used in our prior national representative survey (the first MABAT youth survey) carried out during 2003–4 [18]. In order to test SCOFF validity, we tested the relationships between the SCOFF score and two other variables: (1) Weight dissatisfaction: *r* = 0.31, *p* < .001, (2) Wanting to go on a diet: *r* = 0.26, *p* < .001. These suggest that SCOFF score is strongly related to other reported items and implies convergent validity.

Dinner routine was determined by the question “Where did you eat dinner yesterday?” with the following response options: *at home with parents/family; at home alone; at school, a friend’s house, a restaurant/cafeteria; I did not eat yesterday.*

### Statistical analysis

The data were analyzed using the SAS 9.1.4 program. Descriptive analyses were performed on sex, age, SES of the school, BMI categories, dinner options, and each of the five questions comprising the SCOFF scale among Jews and Arabs (Table 1). A likely case of DE was computed according to Morgan et al. [31] (i.e., more than two affirmative responses to the SCOFF items). First, the associations between the dependent variable of DE (i.e., > 2 affirmative SCOFF items) and the independent variables of sex, age, SES of the school, BMI categories, and dinner options were calculated separately using chi square statistics among Jewish and Arab adolescents (Table 2). Second, the same independent variables were inserted into two multivariate logistic regression models (one for Arabs and one for Jews) with DE (i.e., > 2 affirmative SCOFF items) as an outcome (Table 3). Statistically significant level was determined as *p* < .05.

**Table 1:** Descriptive characteristics of the study population by population group, The 2nd National Health and Nutrition youth survey, 2015–2016

	Jews *N* = 3251 n(%)	Arabs *N* = 1675 n(%)	chi-square, *p* value
**Sociodemographic characteristics:**			
Sex			**32.15,*p*** **< 0.001**
Females	1594(49.03)	964(57.55)	
Males	1657(50.97)	711(42.45)	
Age (years)			2.24, *p* = 0.13
11–14	1596(49.09)	860(51.34)	
15+	1655(50.91)	815(48.66)	
BMI category			**21.10,*p*** **< 0.001**
Underweight	67(2.38)	28(1.78)	
Healthy weight	1958(69.48)	1015(64.44)	
Overweight	528(18.74)	323(20.51)	
Obese	265(9.40)	209(13.27)	
SES of school			**91.11,*p*** **< 0.001**
Low	1600(49.22)	1064(63.52)	
High	1651(50.78)	611(36.48)	
**Disordered Eating (DE) by SCOFF** [31] **items:**			
1. Do you make yourself sick because you feel uncomfortably full?	321(10.57)	137(10.16)	0.162, *p* = .69
2. Do you worry you have lost control over how much you eat?	1208(37.53)	623(37.31)	0.023, *p* = .88
3. Have you ever lost more than 3 kg in weight over a three-month period?	1207(37.24)	756(45.60)	**31.89,*p*** **< .001**
4. Do you believe yourself to be fat when others say you are too thin?	818(25.37)	499(30.01)	**11.97,*p*** **< .001**
5. Would you say that food dominates your life?	942(29.07)	531(31.78)	**3.84,*p*** **= .050**
Likely case of DE (> two positive responses)	635(19.53)	421(25.13)	**20.59,*p*** **< .001**
**Where did you eat dinner last night?**			**141.53,*p*** **< .001**
At home with parents/family	1988(64.03)	1019(69.23)	
At home alone	388(12.50)	150(10.19)	
At school/friend’s house/restaurant/cafeteria	531(17.10)	119(8.08)	
Did not eat dinner	162(5.22)	174(11.82)	

Notes

Significant associations (*p* < 0.05) appear in bold

Underweight: BMI percentile< 3%, Healthy weight: 3% ≤ BMI percentile< 85%, Overweight: 85% ≤ BMI percentile< 97%, Obese: BMI percentile≥97%

SES: The school’s affiliated welfare level according to the Ministry of Education

Likely case of DE is defined as a total SCOFF [31] score of more than two affirmative responses

**Table 2:** Rates of Disordered Eating (DE) by sociodemographic variables, BMI categories, and dinner options among Jewish and Arab adolescents - Results of univariate analyses, The 2nd National Health and Nutrition youth survey, 2015–2016

	Jews *N* = 3251		Arabs *N* = 1675	
	Rates of DE n(%)	Differences in rates across categories chi-square, *p* value	Rates of DE n(%)	Differences in rates across categories chi-square, *p* value
**Sex**		**χ**^**2**^ **= 170.73,*p*** **< .001**		**χ**^**2**^ **= 52.71,*p*** **< .001**
Females	459(28.80)		306(31.74)	
Males	176(10.62)		115(16.17)	
**Age (years)**		**χ**^**2**^ **= 29.85,*p*** **< .001**		χ^2^ **=** 3.31, *p* = .069
11–14	250(15.66)		200(23.26)	
15+	385(23.26)		221(27.12)	
**BMI category**		**χ**^**2**^ **= 71.27,*p*** **< .001**		**χ**^**2**^ **= 22.54,*p*** **< .001**
Underweight & Healthy weight	305(15.06)		224(21.48)	
Overweight	149(28.22)		101(31.27)	
Obese	80(30.19)		71(33.97)	
**SES of school**		**χ**^**2**^ **= 10.42,*p*** **= .001**		χ^2^ **=** 0.97, *p* = .32
Low	349(21.81)		259(24.34)	
High	286(17.32)		162(26.51)	
**Where did you eat dinner last night?**		**χ**^**2**^ **= 27.77,*p*** **< .001**		**χ**^**2**^ **= 23.33,*p*** **< .001**
At home with parents/family	349(17.56)		225(22.08)	
At home alone	78(20.10)		45(30.00)	
At school/friend’s house/restaurant/cafeteria	120(22.60)		41(34.45)	
Did not eat dinner	54(33.33)		63(36.21)	

Notes

DE is defined as a total SCOFF [31] score of more than two affirmative responses

Significant associations appear in bold (*p* < 0.05)

Underweight: BMI percentile< 3%, Healthy weight: 3% ≤ BMI percentile< 85%, Overweight: 85% ≤ BMI percentile< 97%, Obese: BMI percentile≥97%

SES: The school’s affiliated welfare level according to the Ministry of Education

**Table 3:** Multivariate logistic regression models to predict Disordered Eating (DE) by sociodemographic variables, BMI categories, and dinner options among Jewish and Arab adolescents – The 2^nd^ National Health and Nutrition youth survey, 2015-2016

Characteristic	Jews *N*=3,251		Arabs *N*=1,675	
	OR	95%CI	OR	95%CI
**Sex**				
Females	**3.74**	[2.99:4.67]	**2.82**	[2.13:3.74]
Males	Ref		Ref.	
**Age (years)**				
11-14	Ref.		Ref.	
15+	**1.42**	[1.15:1.75]	**1.41**	[1.09:1.81]
**BMI category**				
Under & Healthy weight	Ref.		Ref.	
Overweight	**2.39**	[1.81:3.18]	**1.80**	[1.33:2.44]
Obese	**2.55**	[1.74:3.72]	**2.38**	[1.63:3.46]
**SES of school**				
Low	Ref		Ref.	
High	1.15	[0.93:1.41]	0.89	[0.69:1.16]
**Where did you eat dinner last night?**				
At home with parents/family	Ref.		Ref.	
At home alone	1.13	[0.83:1.55]	**1.52**	[1.01:2.27]
At school/friend’s house/ restaurant/cafeteria	**1.37**	[1.05:1.79]	**1.74**	[1.13:2.67]
Did not eat dinner	**1.91**	[1.29:2.81]	**1.59**	[1.10:2.29]

**Notes:**

DE is defined as a total SCOFF [32] score of more than two affirmative responses

Underweight: BMI percentile<3%, Healthy weight: 3%≤BMI percentile<85%, Overweight: 85%≤BMI percentile<97%, Obese: BMI percentile≥97%

SES: The school’s affiliated welfare level according to the Ministry of Education

Significant associations appear in bold (*p*<0.05)

Overall percentage of the explained variance: Arabs: 0.68 (95%CI=0.64-0.72), Jews: 0.71 (95%CI=0.68-0.74)

## Results

The compliance of the schools was very high. In the Jewish sector, out of 156 schools sampled, 142 schools participated. Only 14 schools (9%) declined to participate in the survey, and they did not differ from the schools that did participate in terms of their welfare level. In the Arab sector, all schools approached (78) agreed to take part, with the exception of three schools (3.8%).

Out of a total of 7029 middle and high-school children who were enrolled in all the classes surveyed, 5588 questionnaires were received (79.5% response rate). Of them, 353 questionnaires were omitted because of partial data or lack of reliability in the answers (e.g., self-reported age not between 11 and 19 years). In this study, we included only adolescents who had a valid SCOFF score (4926 out of 5235, 94.1%).

This study included a total of 4926 participants comprising 1594 Jewish girls, 964 Arab girls, 1657 Jewish boys, and 711 Arab boys. As can be seen in Table 1, rates of positive responses to three SCOFF items were significantly higher among Arab than Jewish adolescents: food controls life (32% Arabs compared to 29% Jews, *p* = 0.050); losing more than 3 kg in 3 months (46% Arabs compared to 37% Jews; *p* < 0.001), and seeing oneself as fat (30% Arabs compared to 25% Jews, *p* < 0.001). Table 1 also shows that 19.53% of Jews and 25.13% of Arabs had DE (or SCOFF > 2). We calculated overall country prevalence (not shown in a table) and we found that 20.95% of adolescents in Israel had DE (stratified by gender: 29.74% of girls and 12.15% of boys; stratified by age group: 17.59% of younger adolescents and 23.52% of older adolescents).

There were also significant differences between Arabs and Jews in their response to the question, “Where did you eat dinner yesterday” (chi^2^ = 141.53, *p* < .001). Post-hoc chi-square analyses were conducted in order to specify the dinner options that differed among Jews and Arabs. Arab adolescents were more likely than Jewish adolescents to eat yesterday’s dinner at home with parents/family (chi^2^ = 10.75, *p* = .001) or not to eat dinner at all (chi^2^ = 63.27, *p* < .001). Jewish adolescents were more likely than Arab adolescents to eat yesterday’s dinner at home alone (chi^2^ = 5.37, *p* = .021) or to eat it out of the home (chi^2^ = 67.65, *p* < .001).

Table 2 presents the results of univariate analyses of the association between DE (or SCOFF > 2) and sociodemographic characteristics, BMI category, and dinner options. Girls (Jewish: 29%; Arabs: 32%, chi^2^ = 2.49, *p* = .11) were more likely to report DE relative to boys (Jewish: 11%; Arabs: 16%. chi^2^ = 14.23, *p* = .001). Disordered eating was more prevalent among older (15 and above) adolescents (Jews: chi^2^ = 29.85, *p* < .001; Arabs: chi^2^ = 3.31, *p* = .069) and among overweight/obese (vs. healthy weight/underweight) adolescents (Jews: chi^2^ = 71.27, *p* < .001; Arabs: chi^2^ = 22.54, *p* < .001). Among Jews, DE was also associated with lower school SES (chi^2^ = 10.42, *p* = .001).

**Fig. 1:**
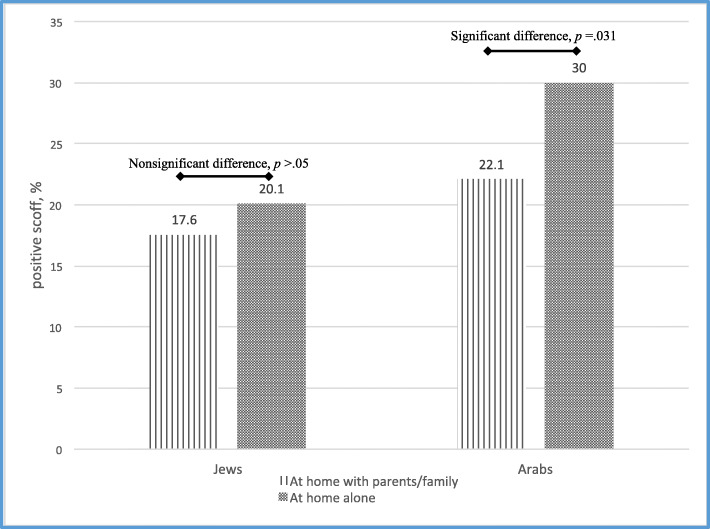
Eating dinner alone (vs. with parents/family) and disordered eating (DE) among Jewish and Arab adolescents in Israel, The 2nd National Health and Nutrition youth survey, 2015–2016.. Note: 30% of Arabs (20% of Jews) who ate dinner alone and 22% of Arabs (18% of Jews) who ate dinner with parents/family had disordered eating (DE) according to the SCOFF questionnaire [31]

There was a significant association between dinner options and DE, with the highest rates of DE among adolescents who did not eat dinner yesterday (Jews: 33%, Arabs: 36%), and the lowest rates among adolescents who reported eating yesterday’s dinner at home with parents/family (Jews: 18%, Arabs: 22%). Post-hoc chi-square analyses revealed (Fig. 1) that among Jews, rates of DE did not differ among adolescents who reported eating yesterday’s dinner alone or eating it with parents/family (20.10 and 17.56%, respectively; chi^2^ = 1.42, *p* = .20). However, among Arabs, rates of DE were significantly higher among adolescents who reported eating yesterday’s dinner alone relative to eating it with parents/family (30.00 and 22.08% respectively; chi^2^ = 4.61, *p* = .031).

Multivariate logistic regression models (Table 3) were performed separately for Arabs and Jews. Among both Jewish and Arab adolescents, respectively, being female (OR = 3.74, 95%CI = [2.99–4.67]; OR = 2.82, 95%CI = [2.13–3.74]), older (OR = 1.42, 95%CI = [1.15–1.75]; OR = 1.41, 95%CI = [1.09–1.81]), and either overweight (OR = 2.39, 95%CI = [1.81–3.18]; OR = 1.80, 95%CI=[1.33–2.44]) or obese (OR = 2.55, 95%CI = [1.74–3.72]; OR = 2.38, 95%CI = [1.63–3.46]), were associated with DE. Compared to eating yesterday’s dinner at home with parents/family, eating it at home alone was associated with a higher likelihood of DE only among Arabs (Jews: OR = 1.13, 95%CI = [0.83–1.55]; Arabs: OR = 1.52, 95%CI = [1.01–2.27]). In addition, both eating yesterday’s dinner at school/a friend’s/restaurant/cafeteria (Jews: OR = 1.37, 95%CI = [1.05–1.79]; Arabs: OR = 1.74, 95%CI = [1.13–2.67]) and not eating it at all (Jews: OR = 1.91, 95%CI = [1.29–2.81]; Arabs: OR = 1.59, 95%CI = [1.10–2.29]) were independently associated with a higher likelihood of DE among both ethnic groups. The *p*-values of both logistic regressions were *p* < .001, and the overall percentage of the explained variance the models captured was 0.68 (95%CI = 0.64–0.72) for Arabs and 0.71 (95%CI = 0.68–0.74) for Jews.

## Discussion

This study, which focused on DE and family dinners among Arab and Jewish middle and high-school children in Israel by using nationally representative data, brings to light several unique findings. First, about one out of every five Jewish adolescents and one out of every four Arab adolescents in Israel reported having DE, according to the SCOFF scale. Second, while Arab and Jewish girls reported comparable rates of DE (Jews: 29%; Arabs: 32%), Arab boys reported a significantly higher prevalence of DE compared to Jewish boys (Jews: 11%; Arabs: 16%). Third, eating yesterday’s dinner at home with parents/family members was found to be slightly more common among Arab than Jewish adolescents. Fourth, eating dinner with parents/family members seems to act as a protective factor against DE among Arab and Jewish adolescents in Israel.

To our knowledge, the current study is the first nationally representative assessment of DE among Arab and Jewish adolescents in Israel. The rates reported previously by Kaluski et al. [18] (using data from the first MABAT youth study) included only girls and were based on only four out of the five SCOFF items. Kaluski et al. [18] defined DE as consisting of more than one affirmative SCOFF response, and not as consisting of more than two affirmative responses, as suggested by Morgan et al. [31] Furthermore, Latzer et al. [21], who also reported rates of DE in Israel, concentrated solely on girls, using the Eating Disorder Inventory (EDI) [37] with a non-representative sample and without anthropometric measurements. It is important to note that the rates of DE reported in our study are similar to those reported by other Western countries using the SCOFF questionnaire. For example, German and Finnish studies [12, 38] among large adolescent samples, reported that 24–29% of girls and 15–16% of boys showed at least one symptom of eating disorders.

Our findings suggest slightly higher rates of DE among Arab adolescents compared to Jewish adolescents in Israel, with Arab boys reporting higher rates than Jewish boys, and Arab girls reporting similar rates to Jewish girls. This finding is not surprising, as previous reports had already suggested high rates of DE among Arab youth in Israel [18, 21]. This result is in line with some prior research about DE among ethnic minority youth in the USA, including Hispanic and American Indian youth who reported higher rates of DE relative to white youth [10, 11]. One explanation for this finding could be that youth of ethnic minorities, living in a dominant Western culture, are likely to adopt ideas that stress the importance of body shape and weight (e.g., the thin ideal standard of beauty) [9], as they perceive these ideas as being a way of acclimating to the dominant culture. Relatedly, a study about Muslim-Australian women suggested a positive link between adopting Western values and DE [39]. Nevertheless, some prior results have demonstrated that ethnicity may protect against the development of eating disorder symptoms [40]. Future studies are warranted to understand the exact mechanism which encourages youth of ethnic minority groups to adopt these ideas about the importance of body weight and shape.

Participants were asked to indicate where and with whom they had eaten dinner the day before. The prevalence of home family dinners was significantly higher among Arabs (69%) relative to Jews (65%), but the difference was negligible, suggesting that patterns of family dinners are quite similar among Arabs and Jews. However, interesting differences between Arabs and Jews were found in the alternatives to home family dinners, as when not eating dinner together with parents and family, Arabs were more likely not to eat dinner at all (Arabs:12% Jews: 5%), and Jews were more likely to eat out of the home (Arabs: 8%, Jews: 17%). More research is warranted to specify the reasons behind these differences in dinner patterns among Arab and Jewish adolescents (e.g., differences in SES, family structure, traditions, values).

Multivariate analyses stressed that among both groups (i.e., Arabs and Jews) female gender, older age, and higher BMI were linked with a higher likelihood of DE. Furthermore, relative to home family dinners, all other dinner options (i.e., not eating dinner or eating out of the home among Arabs and Jews, and eating at home alone among Arabs) acted as risk factors for DE. These findings are in line with the literature, as several studies have already demonstrated that male gender, younger age, lower BMI, and frequent family meals can be protective against DE among youth [28]. In addition, Fiese et al. [41] stress that family mealtime is a household routine that provides stability and predictability for children and is related to several important positive child health outcomes, including a lower prevalence of DE. Family meals can serve as opportunities for parents to demonstrate healthy eating patterns, potentially influencing children’s DE attitudes and behaviors [42]. Furthermore, family dinners may provide opportunities for parents to set examples for healthy eating practices and to expose children to a variety of foods. In addition, the time when all family members are sitting around the table can be used in order to engage in a group discussion during which parents gain exposure to adolescents’ life, while also establishing routines and strengthening family connections [24].

Interestingly, eating dinner at home alone (vs. the home family dinner) acted as a risk factor for DE only among Arab, but not among Jewish, adolescents. It is possible to speculate that among some families, parents may have been involved in the child’s meal planning (e.g., by providing him/her with a plate of food), even when the child ate dinner at home alone. Therefore, it could be that among some participants (e.g., Jewish adolescents), eating at home alone was not necessarily linked with a higher risk for DE. Additionally, among both ethnic groups, not eating dinner at all, as well as eating dinner out of the home (vs. the home family dinner), were both related to a higher risk for DE. It is possible that when children eat dinner outside of the home or do not eat dinner at all, parents have fewer opportunities to be vigilant about their children’s eating patterns, as well as a lessened capability to have an impact on their food-related attitudes and behaviors [42]. Indeed, prior studies have suggested that both skipping meals [43] and eating out of the home [44, 45] are linked with weight gain and unhealthful dietary intake, probably due to a lack of parental involvement in the child’s eating habits on those occasions. In contrast, when the child eats at home alone, parents may potentially still be involved in the child’s meal (e.g., via the preparation of the food), and therefore have an impact on his/her eating patterns. It is thus important to look not only at the frequency of family meals, but also at the alternative routines that parents adopt in order to remain vigilant over their child’s eating patterns.

The present study used the SCOFF questionnaire to assess DE prevalence. Several other questionnaires are available for the screening of eating disorders and DE (e.g., EAT-26 [46], EDI [21]), and the prevalence rates of DE among youth might vary considerably depending on the questionnaire and the methods used to obtain the data. The SCOFF is probably the most promising short questionnaire to use in community samples, mainly because it is a brief self-report scale with only five items [47]. Confirmatory factor analyses [48] that assessed the SCOFF in comparison with health examinations among adolescents indicated that the SCOFF is a useful tool for the detection of DE. Relatedly, the third item of the SCOFF assesses for losing more than one stone (=6.35 kg) in weight over a three-month period, while in the design of the current study, a decision was made to be consistent with the first MABAT study [18] by asking participants about losing more than half a stone (=3 kg) in weight. The literature suggests that among adolescents, the third SCOFF item, which asks about weight loss of one stone, fails to provide accurate information in terms of risks for eating disorders [32, 49]. For example, a recent study [49], examining the SCOFF questionnaire among high school population in Ohio, reports that when examining the correlation of each of the five SCOFF items with the summed score scale, the original third item of the SCOFF has the lowest correlation coefficient. In addition, this same study suggests that the original third item of the SCOFF has the worst ability to discriminate risk for eating disorders (relative to the other SCOFF items).

Findings may have several important implications for health policy. Importantly, given that our data indicated high rates of DE among both Arab and Jewish adolescents in Israel, there is a need to devote funding to DE prevention efforts (e.g., through the dedicated training of healthcare providers working with adolescents) [50]. Furthermore, our findings about the link between family dinners and DE may suggest that possible programs to prevent and reduce DE should be conducted not only among adolescents but also among their family members, with a particular emphasis on educating parents of both Arab and Jewish youth regarding the risks associated with low frequency of family meals.

### Strengths and limitations

This study marks a first step in addressing an important issue for the health and well-being of Arab and Jewish adolescents. To our knowledge, this is the first study to investigate DE and dinner options among Arab and Jewish adolescents in Israel. The strengths of the current study lie in the high response rate and the large representative sample that included adolescents from both Arab and Jewish schools. Additionally, the BMI was calculated based on measured height and weight by trained personnel, providing a reliable and valid estimate of BMI status. While this study has multiple strengths, it also has several limitations. First, the cross-sectional nature of this study makes it difficult to determine the direction of influence and whether a higher frequency of eating alone contributes to, or results from, more severe DE symptoms. Second, we assessed where and with whom dinner was eaten, but we did not have information about breakfast and lunch [23]. Third, the majority of the data in this study were self-reported and therefore subject to reporting bias, including a social desirability response bias. Fourth, the MABAT Youth Survey is a school-based study and does not include children who are not in school or children who are in private, independent, or boarding schools, including the ultra-Orthodox (Haredi) sector. Fifth, the SES variable was defined ecologically according to the Ministry of Education’s classification of the welfare level of the school. Last, dinner options were reported only in regard to the previous day. Nevertheless, studies suggest that data reported about the previous day may be quite accurate relative to other reporting methods (e.g., reporting about weekly frequency of family meals), because they (the former) are less subject to recall bias [51]. Last, positive and negative predictive values of the SCOFF questionnaire were not examined in the current study. However, a recent systematic review and meta-analysis [32] suggests that the SCOFF is moderately helpful in detecting and ruling out the presence of an eating disorder.

## Conclusions

In the current study, we analyzed nationally representative data of middle and high-school children in Israel in order to examine the prevalence of family dinners and DE among Arab and Jewish adolescents, and in order to understand the relations between eating dinner with the family and DE. The findings suggest several differences in family dinner patterns and rates of DE among Arabs and Jews, and imply that family dinners may act as a protective factor against DE. Future work should look at additional variables regarding family meals, such as the quantity and type of food consumed when eating alone (as opposed to the food consumed during family meals), the number of participants, the identity of the family participants, and the time spent sitting together at the table, as well as other family factors such as perceived family atmosphere, family cohesion, and family stability. Future studies should investigate these findings using a longitudinal or case-control design in order to examine the occurrence of family meals, both before and after DE attitudes and behaviors develop, among Arab and Jewish youth in Israel and around the Western world. Some preliminary evidence [52] suggests that designated interventions can improve the frequency and quality of family meals. Thus, it would be important to conduct research that explicitly examines (e.g., using a randomised clinical trial) whether interventions tailored to improve the frequency and quality of family meals (e.g., among populations at risk, such as Arab families in Israel) can ultimately reduce DE attitudes and behaviors. Given the importance of DE as an obstacle in the health of a population, we recommend that government agencies fund a research agenda along the lines we have outlined.

## Data Availability

Data will be shared upon request.
